# Neurocognitive status and risk of mortality among people living with human immunodeficiency virus: an 18-year retrospective cohort study

**DOI:** 10.1038/s41598-021-83131-1

**Published:** 2021-02-12

**Authors:** Zaeema Naveed, Howard S. Fox, Christopher S. Wichman, Morshed Alam, Pamela May, Christine M. Arcari, Jane Meza, Steven Totusek, Lorena Baccaglini

**Affiliations:** 1 Department of Epidemiology, College of Public Health, University of Nebraska Medical Center, 984355 Medical Center, Omaha, NE 68198-4395 USA; 2 Department of Neurological Sciences, University of Nebraska Medical Center, Omaha, NE USA; 3 Department of Biostatistics, University of Nebraska Medical Center, Omaha, NE USA

**Keywords:** Neuroscience, Neurology, Risk factors, Virology, Retrovirus, Viral epidemiology, Diseases, HIV infections

## Abstract

HIV-related neurocognitive impairment (NCI) may increase the risk of death. However, a survival disadvantage for patients with NCI has not been well studied in the post-combination antiretroviral therapy (cART) era. Specifically, limited research has been conducted considering the reversible nature and variable progression of the impairment and this area demands further evaluation. We performed multivariable Cox proportional hazards modeling to assess the association between baseline NCI (global T scores) and mortality. A joint modeling approach was then used to model the trajectory of global neurocognitive functioning over time and the association between neurocognitive trajectory and mortality. Among the National NeuroAIDS Tissue Consortium’s (NNTC) HIV-infected participants, we found a strong negative association between NCI and mortality in the older age groups (e.g., at age = 55, HR = 0.79; 95% CI 0.64–0.99). Three neurocognitive sub-domains (abstraction and executive functioning, speed of information processing, and motor) had the strongest negative association with mortality. Joint modelling indicated a 33% lower hazard for every 10-unit increase in global T scores (HR = 0.67; 95% CI 0.56–0.80). The study identified older HIV-infected individuals with NCI as a group needing special attention for the longevity of life. The study has considerable prognostic utility by not only predicting mortality hazard, but also future cognitive status.

## Introduction

Since the outset of the human immunodeficiency virus (HIV) epidemic, HIV-associated neurocognitive disorders (HAND) have been prevalent in infected populations, ranging from subtle neuropsychological impairments to profoundly disabling HIV-associated dementia (HAD)^1^. The advent of combination antiretroviral therapy (cART) has transformed HIV infection from a deadly acute disease to a chronic tractable condition by effective management of HIV viremia and enhanced immune function^2^. Yet, despite the widespread use of cART, HIV-associated neurocognitive impairment (NCI) and brain injury persist with a change in phenotype and pattern. There has been a significant decrease in HAD in the cART era. Nevertheless, less severe forms of HAND continue to have a prevalence of 20–50%^3,4^. The pattern of NCI also differs between the two eras. In the pre-cART era impairment in motor skills, cognitive speed, and verbal fluency were more common, whereas in the cART era memory and executive function impairment are more prominent^1,3,5^.

HIV enters the brain early in its course by crossing the blood–brain barrier inside migrating monocytes and lymphocytes. Infected monocytes are converted to perivascular macrophages that express neurotoxic molecules leading to increased blood–brain barrier permeability. Neuronal damage and death are ensued both by direct viral proteins interaction and indirect inflammatory response mounted by inflammatory cells against the viral proteins^6,7^. The brain alterations during early HIV infection have also been validated by neuroimaging^8^. In the past, subcortical regions of the brain were thought to be primarily infected by HIV, giving rise to subcortical dementia. However, heterogeneous findings from both neuropsychological and neuroimaging studies have now recognized the cognitive impairment to be present across various brain regions and cognitive domains^9^. The exact mechanism of persistence of milder forms of impairment is not clear; though, two potential explanations may include the lingering consequences of advanced immunosuppression during the early stages of the disease (before initiation of cART) and ongoing viral replication within the brain, even when systemic viral suppression has been achieved^10,11^.

NCI progression is highly variable, with individuals displaying considerable recovery of cognitive functions, worsening of impairment, static impairment, or a fluctuating course^12^. Understanding the consequences of HIV associated NCI is vital because even in milder forms, it is associated with lower medication adherence, a decreased ability to perform the daily tasks, poorer quality of life, and difficulty obtaining employment^13^. Moreover, HIV infected individuals with mild cognitive impairment may have an increased risk of dementia and death^14,15^. Although HIV related morbidity and mortality have decreased over time, people living with HIV continue to face an increased risk of mortality compared to the non-infected counterparts, even among those with a successful response to cART^16,17^. There are several well-established predictors of mortality in HIV^18–20^; however, limited research has been conducted to investigate the association between NCI and mortality in HIV infected people. In the pre-cART era, NCI ascertained through a comprehensive battery of neuropsychological tests was found to be an independent risk factor of death^15,21,22^. A recent cross-sectional study with hospitalized HIV infected patients as the study sample found higher inpatient mortality among those who had been diagnosed with HIV associated NCI compared to those who had not^23^. Three other studies conducted in the cART era found a positive association between NCI and mortality but were limited to participants with advanced HIV infection or severe cognitive disorders only^24–26^. Banerjee et al. recently reported NCI to be an independent prognostic marker of mortality in an HIV infected cognitive cohort^27^. The study used the HIV-Dementia scale (HDS) to assess NCI. However, studies have demonstrated inconsistent results pertaining to the ability of HDS to detect subtle types of NCI^28,29^.

Apart from the limited research on the association between NCI and mortality, all the previous studies have examined cognitive impairment at a single time-point (i.e., baseline). As it is likely that cognitive status changes over time, it is vital to account for this variability in relation to mortality. A survival disadvantage for patients with NCI has not been well studied in the cART era, particularly taking into consideration the reversible nature of the impairment and demands further evaluation. The present study aims to fill the research gap by examining the association between baseline neurocognitive status as well as longitudinal changes in neurocognitive status and mortality in a diverse HIV-infected sample. We hypothesize that NCI and its progression increase the hazard of death in HIV patients either independently or in association with specific patient-related factors.

## Methods

### Data source and participants

The National NeuroAIDS Tissue Consortium (NNTC) database was used to investigate the association between neurocognitive status and mortality in HIV patients. NNTC is an ongoing, prospective observational study established in 1998 with the primary aim of collecting, storing, and distributing samples of central and peripheral nervous system tissue, cerebrospinal fluid, blood, and other organs collected from HIV positive and negative patients for research purposes^30^. Adult participants with advanced HIV disease willing to participate in a post-mortem organ donation program were recruited at one of the four participating sites: Texas NeuroAIDS Research Center (University of Texas Medical Branch, Galveston), California NeuroAIDS Tissue Network (University of California, San Diego), National Neurological AIDS Bank (University of California, Los Angeles) and Manhattan HIV Brain Bank (Mount Sinai Medical Center, New York). Participants were volunteers recruited from clinics, hospitals, and local communities into a longitudinal observational study with detailed neurologic and neuropsychological evaluations at 6-, 12- or 24-month intervals depending on the clinical judgment of a participant’s health.

Variables such as demographics, medication history (ARV and others), cerebrospinal fluid, blood, plasma, and urine laboratory testing for HIV specific and ancillary markers, comorbidities and substance use were collected at baseline and during the longitudinal phase^31,32^.

For the analysis, we included participants enrolled between January 2000 to November 2017, with complete baseline data on the variables of interest, and with at least two follow-up visits (n = 1,325). Seventy-seven participants did not have information available on neurocognitive status at the baseline and were excluded (n = 1,248). Further exclusions were made based on missing baseline information on covariates of interest. The reporting of this observational study has been guided by the STROBE instrument.

### Variables

The primary outcome was time to event (death). The primary exposures were neurocognitive status at baseline (for Cox proportional hazards modeling) and repeated measures of neurocognitive status (for joint modeling). Neurocognitive status was assessed through a continuous score (demographically corrected T score) derived from a comprehensive neurocognitive test battery comprising of fourteen test scores. The tests with references are given in Table 1. The battery covers seven cognitive domains, including executive functioning, speed of information processing, attention and working memory, learning, memory, verbal fluency, and motor functioning. Raw scores from individual tests were converted to demographically corrected T scores^3^ which were than averaged together to generate the global T score. For descriptive analysis, an impaired neurocognitive status was assigned to those with a global T score value of < 40^33^. The best available normative standards were used, which correct for the effects of age, education, sex, and ethnicity, as appropriate^14^. Based on prior literature and biological plausibility, other groups of variables included in the study were demographic factors (age, education, gender, race, and ethnicity), HIV related factors (disease severity, duration of HIV infection, antiretroviral (ARV) drug use, CD4 nadir, current CD4 cell count, plasma viral loads and CSF viral loads), comorbidities (anemia, cerebrovascular disease, hypertension, diabetes, hyperlipidemia, viral hepatitis, chronic renal disease, chronic obstructive pulmonary disease, AIDS-defining comorbidity, any CNS comorbidity and a composite measure of any non-AIDS defining comorbidity) and substance use (history of alcohol, opiate, hallucinogen, cannabis and cocaine use). The Composite International Diagnostic Interview (CIDI, v2.1) that is consistent with the diagnostic and statistical manual of mental disorders-4th Edition (DSM-IV), was used to measure substance use.

**Table 1:** Neuropsychological test battery used to generate T scores among HIV-infected participants of National NeuroAIDS Tissue Consortium (NNTC).

Cognitive domain	Neuropsychological test
Abstraction/executive functioning	Trail Making Test, Part B^49^
	Wisconsin Card Sorting Test-64, Perseverative Responses^49,50^
Speed of information processing	Wechsler Adult Intelligence Scale-3rd ed. (WAIS-III) Digit Symbol^51^
	WAIS-III Symbol Search^51^
	Trail Making Test, Part A^49^
Attention and working memory	Paced Auditory Serial Addition Task (PASAT) (first channel only)^52^
	WAIS-III Letter Number Sequencing^51^
Learning	Brief Visuospatial Memory Test-Revised (BVMT-R) Total Recall^50,53^
	Hopkins Verbal Learning Test-Revised (HVLT-R) Total Recall^50,54^
Memory	BVMT-R Delayed Recall^50,53^
	HVLT-R Delayed Recall^50,54^
Verbal fluency	Controlled Oral Word Association Test (COWAT-FAS)^49^
Motor	Grooved Pegboard dominant^49^
	Grooved Pegboard non-dominant^49^

### Statistical analyses

Descriptive statistics were generated for categorical (frequencies and percentages) and continuous variables (means, medians, and standard deviations) to assess the overall demographic and clinical characteristics of the sample. Traditional survival analysis was conducted using Cox proportional hazards regression. Kaplan–Meier analysis was used to compute the overall median survival time and to visualize survival time distributions. Univariable Cox proportional hazards models were employed to explore the association between the covariates (primary exposure and potential confounders) and outcome. Furthermore, univariable analyses using one-way analysis of variance (ANOVA) for continuous variables and Cochran Mantel–Haenszel statistics (with modified ridit scores) for categorical variables were conducted to identify the association between primary covariate (neurocognitive status) and other potential covariates. Only those variables associated with, both with the primary outcome and primary covariate at 2-sided α = 0.2, were entered in the multivariable Cox proportional hazards model for further analysis. One-way interactions between the primary predictor and other covariates were also assessed using the proportional hazards model.

For multivariable Cox proportional hazards modeling, a stepwise approach was used. The initial model included neurocognitive status, age, and the interaction term between them. Other potential confounders were then added to the model, and the change in the estimate for the association between neurocognitive status and outcome was recorded. The variable was considered a confounder if the percent change in the estimate of the reduced model compared to the model with the added variable was more than 5%. Finally, HIV duration was forced into the model. The proportional hazards assumption was assessed using the graphical method (log–log survival curve approach) and goodness of fit test (based on martingale residuals). The same model was fitted for all seven domains as sub-analyses. Crude and adjusted hazard ratios (HR) and 95% confidence intervals (95% CI) were reported as the measures of association for the Cox proportional hazards regression analyses. A sub-analysis of participants with complete data on Beck Depression Inventory-II (BDI-II) scores was conducted to investigate potential confounding effects of depressive symptoms.

A joint modeling approach was then used to model the trajectory of global neurocognitive functioning (global T score) over time and the association between neurocognitive trajectory and mortality at the same time. The temporal evolution of longitudinal T score measurements was estimated using a linear mixed-effects model. To model cognitive trajectories, we employed both linear and quadratic functions to observe if the results differed. Residual diagnostics were conducted to check the linear mixed models’ assumptions. The final joint model used a Weibull baseline hazard function and was adjusted for covariates used in the traditional survival analysis. The analyses were performed using SAS 9.4 (SAS Institute, Inc., Cary, NC, USA) software and R software-3.5.0 (JM package).

### Ethical approval

This study was approved by the University of Nebraska Medical Center’s institutional review board (Protocol #282-13-EP). All methods were carried out in accordance with relevant guidelines and regulations. The National NeuroAIDS Tissue Consortium obtained written informed consent for participation, neuro-medical and neuropsychological examinations and for obtaining brain tissue after death for examination and tissue banking. For deceased participants, the decedent's family/legal guardian provided informed consent for tissue donation and release of medical records.

## Results

### Participants’ characteristics

**Figure 1:**
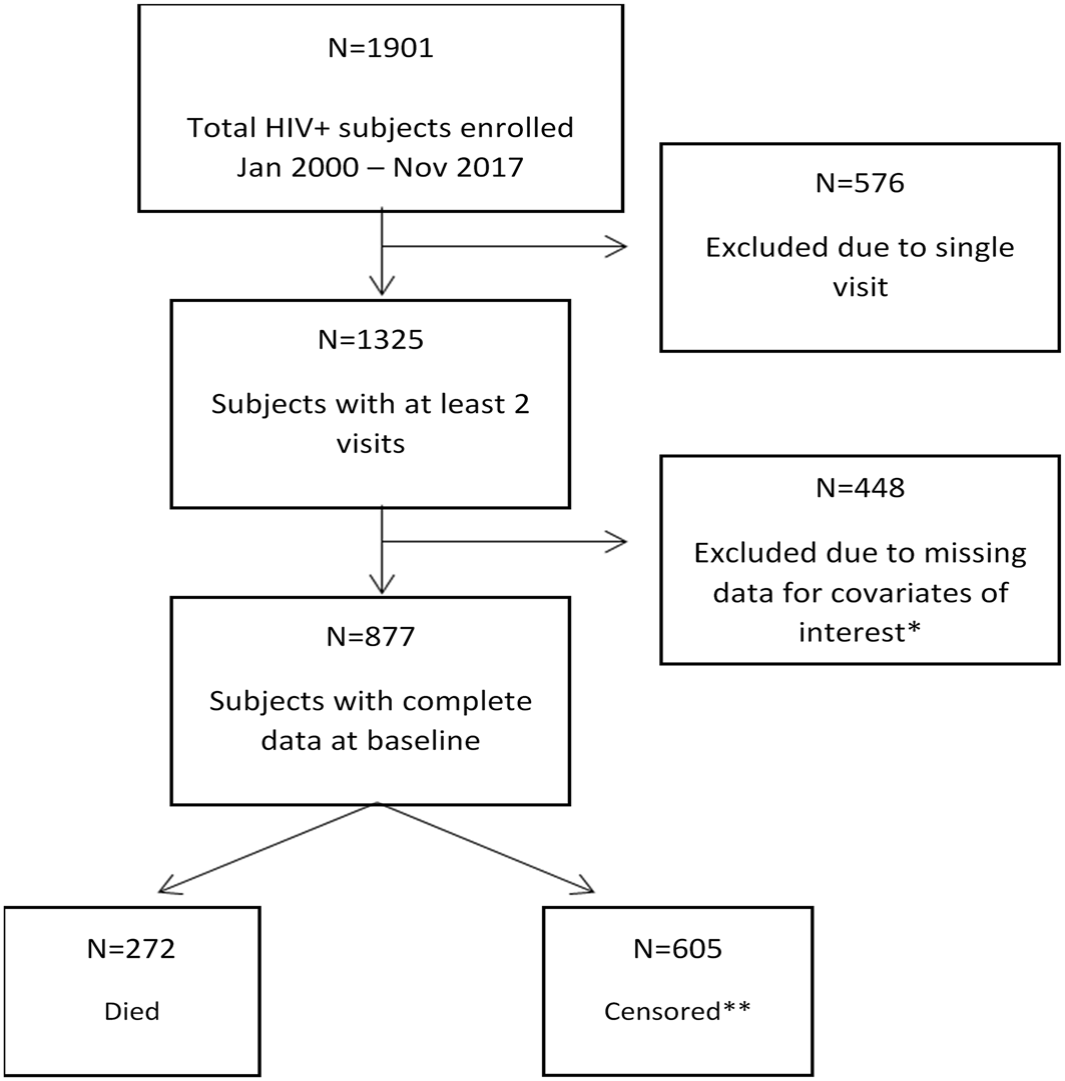
Flow chart of participation. *Descriptive analysis comparing those with missing data for covariates of interest to those with complete data showed no difference in demographic variables and baseline global T scores. **There is a 12% loss to follow-up.

The original enrolled sample (n = 1,901) had a median age of 45 years (IQR = 39–53), and was 81.1% males, 55.8% whites and 73.2% non-Hispanics. Fifty nine percent of the original sample had a global T score above 40. A total of 877 participants were included in the final analyses (Fig. 1). The median age was 45 years (IQR = 40–53), and most participants were males (79.2%), whites (55.8%) and non-Hispanics/Latinos (72.1%). Sixty percent of participants in the analytic sample had a global T score above 40. The median duration of HIV infection was 12.6 years (IQR = 7.4–17.8). At baseline, 78% of participants were on a highly active antiretroviral therapy (HAART) regimen, 13% were on the non-HAART regimen, and the rest 9% were not using any antiretroviral therapy (ART). The median CD4+ cell count was 203.5 cells/µl (IQR = 75–406) and the median Log_10_ viral load was 2.6 (IQR = 1.7–4.2). Table 2 shows the baseline characteristics of participants by neurocognitive status. Most males (80.5%), participants on HAART (81.1%), Blacks (42.0%), and non-Hispanics/Latinos (77.4%) were in the neurocognitively unimpaired category. Furthermore, participants in the unimpaired category were marginally older (mean = 47.1 years) with higher education (mean = 12.3 years), higher hemoglobin levels (mean = 13.4 g/dl), higher CD4 count (mean = 301.9 cells/µl), lower blood Log_10_ viral load (mean = 2.9 copies/ml), longer duration of HIV disease (mean = 13.3 years) and fewer CNS comorbidities (mean = 0.05) compared to those in impaired group. Descriptive statistics for the neurocognitive battery of tests (individual and domain specific T tests) are included in Supplementary Table S1. Participants that died during the follow-up had marginally lower baseline T scores for the domains of abstraction/executive functioning, speed of information processing, verbal fluency and motor and marginally higher T scores for the domains of attention and working memory, learning and working memory compared to those that were censored.

**Table 2:** Participant’s baseline characteristics by neurocognitive status among HIV-infected participants of National NeuroAIDS Tissue Consortium (NNTC).

Variables	Global* T *scores^a^		*p *value
	Unimpaired > 40	Impaired ≤ 40	
	n (%)^b^	n (%)^b^	
Total	527^c^ (60.1)	350^c^ (39.1)	
**Gender**			
Male	424 (80.5)	271 (77.4)	0.2791*
**ARV medication use**			0.0157*
No current ARV	45 (9.1)	29 (8.9)	
Current non-cART	49 (9.8)	59 (18.0)	
Current cART	402 (81.1)	239 (73.1)	
**Race**			0.003*
White	266 (51.9)	211 (61.7)	
Black	215 (42.0)	98 (28.6)	
Other	33 (9.6)	33 (9.6)	
**Ethnicity**			< 0.0001*
Hispanic or Latino	119 (22.6)	126 (36.0)	
Not Hispanic or Latino	408 (77.4)	224 (64.0)	
Hypertension history	121 (29.9)	62 (25.4)	0.2138*
Diabetes history	46 (11.39)	43 (17.7)	0.0242*
Hyperlipidemia history	100 (24.7)	48 (19.6)	0.1291*
Viral hepatitis history	139 (34.5)	104 (42.8)	0.0340*
End stage liver disease history	9 (2.2)	8 (3.3)	0.4157*
Chronic renal disease history	30 (7.5)	17 (7.0)	0.8252*
Cardiac disease history	40 (9.9)	26 (10.7)	0.7611*
Chronic obstructive pulmonary disease	44 (10.9)	24 (9.8)	0.6563*
Cerebrovascular disease history	33 (8.2)	43 (17.7)	0.0005*
Non-AIDS defining cancers	31 (7.7)	15 (6.2)	0.4622*
Any non-AIDS defining comorbidity^d^	287 (70.7)	181 (73.9)	0.3811*
Any CNS comorbidity^e^	29 (5.9)	44 (13.4)	0.0002*
Alcohol use history	224 (61.5)	98 (45.5)	0.0030*
Cannabis use history	151 (41.5)	62 (28.6)	0.0379*
Cocaine use history	198 (54.4)	87 (40.1)	0.0008*
Hallucinogen use history	28 (7.7)	16 (7.4)	0.8883*
Opiate use history	63 (17.3)	42 (19.3)	0.5345*
Sedative use history	38 (10.4)	24 (11.1)	0.8149*
Stimulant use history^e^	86 (23.6)	39 (17.9)	0.1089*

	Mean (SD)	Mean (SD)	
Age (years)	47.1 (10.7)	46.5 (10.6)	0.5779**
Years of education (years)	12.3 (3.6)	12.2 (3.2)	0.5779**
Beck's Depression Inventory (BDI) total score	13.1 (10.1)	15.4 (10.4)	0.0001**
Hemoglobin (g/dl)	13.4 (1.8)	13.2 (1.9)	0.0395**
CD4 Nadir (cells/µl)	138.7 (213.6)	138.1 (210.0)	0.9744**
CD4 cell count (cells/µl)	301.9 (298.8)	273.1 (273.5)	0.1565**
Plasma viral load (log10 copies/ml)	2.9 (1.3)	3.1 (1.4)	0.0561**
Number of non-AIDS defining comorbidities^d^	1.4 (1.3)	1.6 (1.5)	0.2521**
Number of CNS comorbidities^f^	0.05 (0.2)	0.1 (0.4)	< .0001**
Duration of HIV (years)	13.3 (7.4)	12.7 (7.6)	0.1615**
Number of substances used^g^	2.1 (1.7)	1.6 (1.7)	0.0323**

*Cochran Mantel–Haenszel statistics (score = modified ridit). **Analysis of variance.

^a^Scores range between 0 and 100 and higher T scores imply better neurocognitive status.

^b^Column percentages.

^c^Sample size may be lower for variables due to missing data.

^d^Includes history of hypertension, diabetes, viral hepatitis, end-stage liver disease, hyperlipidemia, chronic renal disease, cardiac disease, chronic obstructive pulmonary disease, and cerebrovascular disease.

^e^Defined as use of amphetamines, diet pills, ice, khat, methamphetamine, Ritalin, speed and uppers.

^f^Primary CNS lymphoma, toxoplasma encephalitis, progressive multifocal leukoencephalopathy, CMV ventriculo-encephalitis, cryptococcal meningitis, histoplasma meningitis, coccidiodes meningitis, tuberculous meningitis, syphilitic meningitis, lymphomatous meningitis, and other specific meningitis.

^g^Includes history of alcohol, cannabis, cocaine, hallucinogen, opiate, sedative and stimulant use.

### Survival analysis (Kaplan–Meier method and Cox proportional hazards regression)

**Figure 2:**
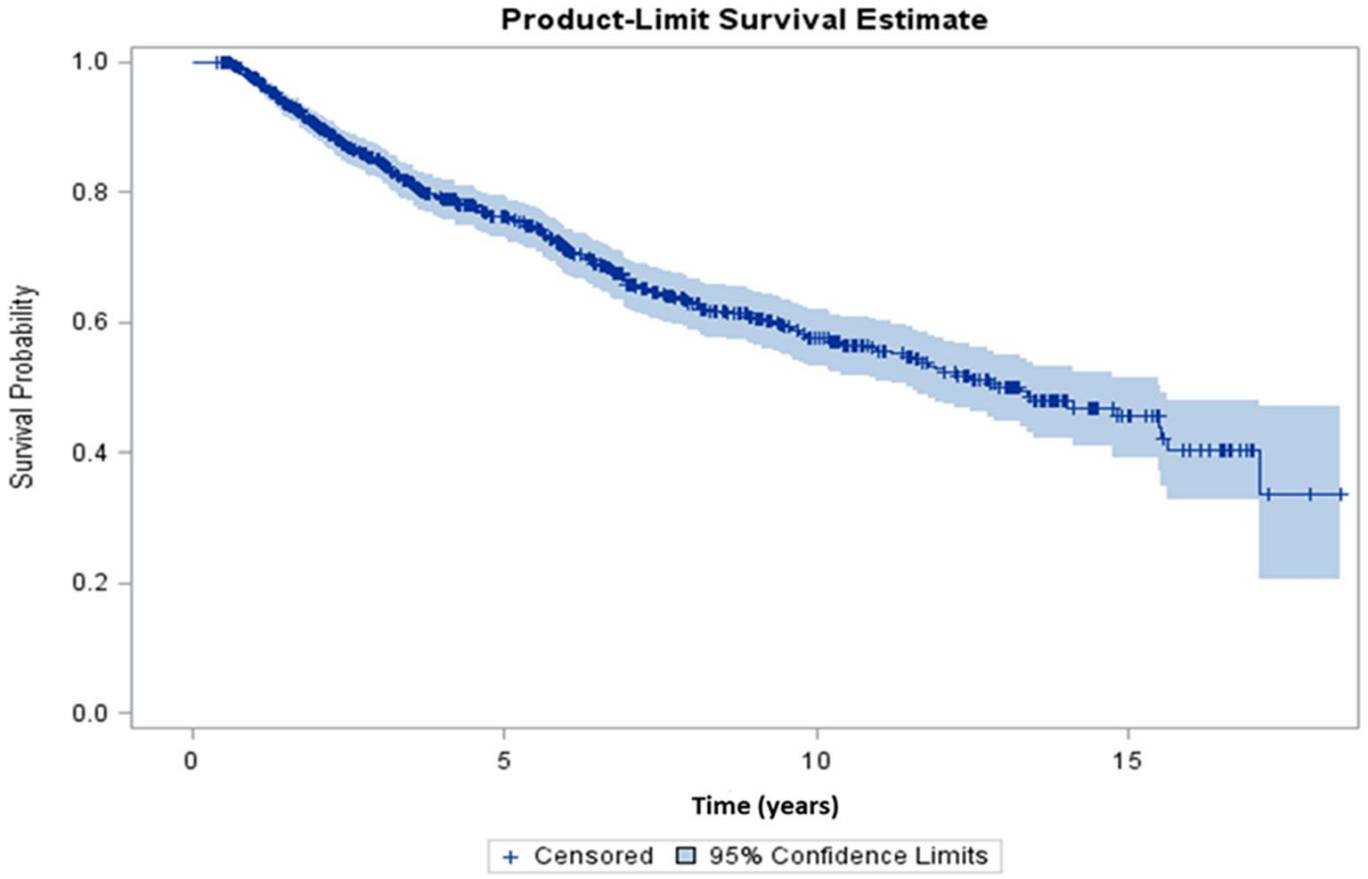
Kaplan–Meier plot for overall survival. Median survival time = 13.2 years (95% CI 11.4–15.5).

**Figure 3:**
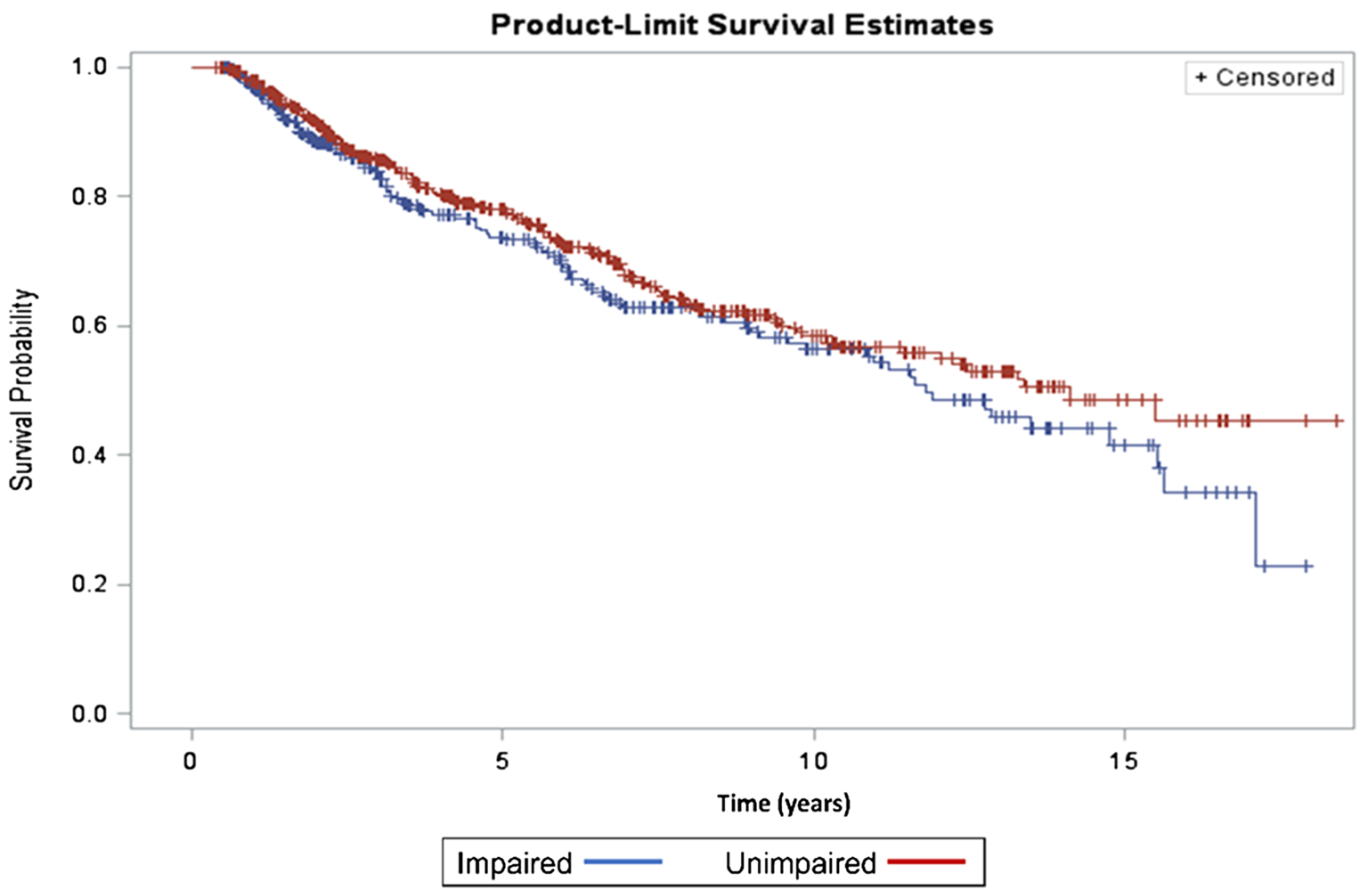
Kaplan–Meier plot stratified by neurocognitive status. Median survival time impaired (global T score ≤ 40) = 11.8 years (95% CI 9.6–15.5). Median survival time unimpaired (global T score > 40) = 14.1 years (95% CI 9.6). *The upper limit of confidence interval for median survival time in unimpaired group is not estimable because of censored data.

The participants were followed for up to 18 years. During the study duration, of the 877 participants, 272 (31%) died. The overall median survival time was 13.2 years (Fig. 2). The survival plot (Fig. 3) stratified by neurocognitive status, exhibited lower median survival time (median = 11.8 years) for neurocognitively impaired participants compared to the median survival time (14.1 years) for unimpaired participants. Unadjusted analyses for interaction and identification of potential confounders showed a significant interaction between age and neurocognitive status in association with mortality. Furthermore, 11 variables (current ARV medication use, ethnicity, history of hyperlipidemia, history of cerebrovascular disease, history of cannabis use, history of cocaine use, history of opiate use, serum hemoglobin, CD4 nadir, plasma viral loads and duration of HIV infection) were assessed as potential confounders in a stepwise approach in the adjusted model. The final multivariable Cox proportional hazards model included duration of HIV infection, ethnicity, serum hemoglobin, plasma viral load and an interaction term between neurocognitive status and age (Table 3). The adjusted model revealed a strong negative association between mortality and NCI in the older age groups. For example, among those who were 55 years old, the hazard of dying for those with higher (10 units) global T score was 0.79 times (21% lower) the hazard for those with lower global T score. However, the association was not evident in the younger age groups. Apart from the interaction term, being non-Hispanic/Latino (HR = 1.38, 95% CI 1.02–1.86) and having a higher baseline log10 viral load (HR = 1.27, 95% CI 1.16–1.39) were associated with a higher hazard of dying whereas, a higher baseline serum hemoglobin was associated with a lower hazard of dying (HR = 0.91, 95% CI 0.85–0.97). Domain-specific Cox proportional hazards modeling (Table 4) showed that T scores for abstraction and executive functioning, speed of information processing, and motor domains, had stronger negative associations with mortality compared to the other domains, particularly among older participants. As seen with the global neurocognitive status, the association between individual neurocognitive domain and mortality was not evident among younger participants.

**Table 3:** Crude and adjusted hazard ratios (HR) for mortality by selected variables among HIV-infected participants of National NeuroAIDS Tissue Consortium (NNTC; n = 877).

Variable (at baseline)	Crude HR (95% CI)	Adjusted HR (95% CI) model^a^
Global T score^b^	0.99 (0.97–1.01)	NA
Age (years)	1.01 (0.99–1.02)	NA
Gender		
Female	Ref	NA
Male	1.20 (0.90–1.61)	
Race		
White	Ref	NA
Black	1.15 (0.89–1.48)	
Other	1.02 (0.63–1.66)	
Duration of HIV infection (years)	1.02 (1.01–1.03)	1.01 (0.99–1.03)
Ethnicity		
Hispanic or Latino	Ref	Ref
Not Hispanic or Latino	1.41 (1.05–1.88)	1.38 (1.02–1.86)
ARV medication use		
No current ARV use	Ref	NA
Current non-cART	0.52 (0.32–0.86)	
Current cART	0.53 (0.37–0.76)	
Serum hemoglobin (g/dl)	0.89 (0.83–0.95)	0.91 (0.85–0.97)
Plasma viral load (log10 copies/ml)	1.28 (1.17–1.39)	1.27 (1.16–1.39)
T scores × age^c^		
35 years	1.13 (0.88–1.46)	1.09 (0.85–1.41)
55 years	0.76 (0.61–9.95)	0.79 (0.64–0.99)
75 years	0.51 (0.31–0.85)	0.58 (0.35–0.97)

^a^Each variable is adjusted for all other variables listed in the column.

^b^Hazard Ratio corresponds to a 10-unit increase in global-t scores.

^c^Interaction term between global T scores and age. Hazard ratio corresponds to a 10-unit increase in global T scores for a given age.

**Table 4:** Neurocognitive domain specific crude and adjusted hazard ratios for mortality by selected variables among HIV-infected participants of National NeuroAIDS Tissue Consortium (NNTC; n = 877).

Variable (at baseline)^a^	Global T score Adjusted HR (95% CI)^a^	Domain specific T score Adjusted HR (95% CI)^a^						
		Abstraction executive functioning	Speed of information processing	Motor	Attention and working memory	Learning	Memory	Verbal fluency
T scores^b^	NA	NA	NA	NA	NA	NA	NA	NA
Age (years)	NA	NA	NA	NA	NA	NA	NA	NA
Duration of HIV infection (years)	1.01 (0.99–1.03)	1.01 (0.99–1.03)	1.01 (0.99–1.03)	1.01 (0.99–1.03)	1.01 (0.99–1.03)	1.01 (0.99–1.03)	1.01 (0.99–1.03)	1.01 (0.99–1.03)
**Ethnicity**								
Hispanic or Latino	Ref	Ref	Ref	Ref	Ref	Ref	Ref	Ref
Not Hispanic or Latino	1.38 (1.02–1.86)	1.37 (1.01–1.84)	1.35 (0.99–1.82)	1.33 (0.99–1.79)	1.29 (0.95–1.74)	1.33 (0.99–1.80)	1.29 (0.96–1.74)	1.35 (1.00–1.81)
Serum hemoglobin (g/dl)	0.91 (0.85–0.97)	0.91 (0.85–0.97)	0.91 (0.85–0.97)	0.91 (0.85–0.97)	0.89 (0.84–0.96)	0.90 (0.84–0.96)	0.90 (0.84–0.96)	0.90 (0.85–0.97)
Plasma viral load (log10 copies/ml)	1.27 (1.16–1.39)	1.27 (1.16–1.39)	1.27 (1.16–1.39)	1.28 (1.16–1.40)	1.28 (1.17–1.41)	1.27 (1.16–1.39)	1.27 (1.16–1.39)	1.25 (1.14–1.37)
**T scores × age**^**c**^								
35 years	1.09 (0.85–0.41)	1.08 (0.90–1.30)	1.09 (0.90–1.33)	0.98 (0.84–1.14)	0.99 (0.80–1.24)	1.11 (0.89–1.37)	1.16 (0.95–1.41)	0.99 (0.82–1.18)
55 years	0.79 (0.64–0.99)	0.83 (0.71–0.97)	0.84 (0.71–0.98)	0.80 (0.68–0.94)	0.95 (0.79–1.14)	0.93 (0.77–1.12)	1.01 (0.85–1.20)	0.89 (0.77–1.03)
75 years	0.58 (0.35–0.97)	0.64 (0.45–0.93)	0.64 (0.43–0.94)	0.65 (0.45–0.94)	0.91 (0.59–1.40)	0.78 (0.50–1.20)	0.88 (0.59–1.32)	0.81 (0.57–1.14)

^a^Each variable is adjusted for all other variables listed in the column.

^b^Hazard Ratio correspond to a 10-unit increase in T scores.

^c^Interaction term between T scores and age. Hazard ratio corresponds to a 10-unit increase in T scores for given age.

A sub-analysis of participants with complete data on Beck Depression Inventory-II (BDI-II) scores showed a similar association between NCI and mortality than without BDI-II in the model. For example, for participants aged 55 years, the adjusted HR was 0.83 (95% CI 0.62–1.12) for the sub-analysis compared to 0.79 (95% CI 0.64–0.99; Table 3) for the model without BDI-II.

### Joint modeling (shared random effects model)

The joint analysis estimated the individual-specific random effects of the longitudinal process simultaneously and specified them as covariates of mortality in the survival process. The linear mixed effect model generated an average regression coefficient of 0.033 (SE = 0.003) (not shown) for the time variable suggesting an increase in global T scores over the study period. The linear slope estimate of the global T score was − 0.39 (SE = 0.089), which indicates a negative association with the risk of mortality (Table 5). The adjusted HR of the slope was 0.67 (95% CI 0.56–0.80), indicating that for every 10-unit increase in global T score, the hazard of death decreased by 33%. The baseline covariates included in the final model (duration of HIV infection, plasma viral load, ethnicity, and serum hemoglobin) were same as those in the traditional Cox regression.

**Table 5:** Results of multivariable joint modeling of repeated measures (Global T score) and survival data among HIV-infected participants of National NeuroAIDS Tissue Consortium (NNTC).

Predictor	β (SE)	Adjusted HR (95% CI)^a^
Intercept	− 2.34 (0.71)	0.09 (0.02–0.38)
Global T score slope	− 0.39 (0.08)	0.67^a^ (0.56–0.80)
Age (years)	0.01 (0.01)	1.01 (0.99–1.02)
Duration of HIV infection (years)	0.02 (0.01)	1.02 (0.99–1.03)
**Ethnicity**		
Hispanic or Latino	Ref	Ref
Not Hispanic or Latino	0.41 (0.15)	1.50 (1.11–2.02)
Serum Hemoglobin (g/dl)	− 0.10 (0.03)	0.90 (0.84–0.96)
Plasma viral load (log10 copies/ml)	0.22 (0.04)	1.25 (1.14–1.36)

^a^Adjusted for all other variables in the model.

^b^Hazard Ratio corresponds to a 10-unit increase in global T scores.

## Discussion

Despite the availability and use of potent antiretroviral medications, mild to moderate HIV-associated NCI is still prevalent^1,3^. Apart from being a disabling consequence of HIV infection, NCI has been found to be an independent predictor of mortality in the pre-cART era^15^. Limited research has been conducted on the association between mortality and NCI in the HAART era, with little consideration given to the substantial variability that the course of NCI over time, in the HIV infected population^12,15^. The present study aimed to examine the association between baseline neurocognitive status as well as longitudinal changes in neurocognitive status and mortality in a diverse HIV-infected patient sample. Our research adds to this limited body of literature by investigating the link between HIV-associated NCI and mortality among HIV-infected participants.

The results of the crude survival analysis demonstrated no association between neurocognitive status and mortality (HR = 0.99). In adjusted analyses we detected a significant interaction between neurocognitive status and age in relation to mortality. Specifically, among older participants, higher global T scores (i.e., better neurocognitive status) were associated with a lower hazard of death, whereas neurocognitive status was not associated with mortality among younger participants. This finding was independent of plasma viral load, ethnicity, duration of HIV infection, and serum hemoglobin as these variables were adjusted for in the final model. Unlike our study, none of the previous studies have reported an interaction between age and neurocognitive status in relation to mortality. However, studies have observed independent associations between age and mortality and age and neurocognitive status^23,34^. Specifically, in the pre-cART era, studies had found NCI to be an independent risk factor for mortality^15^. The association was also found in the cART-era, in a limited number of studies. However, the association was explored either between severe forms of NCI and mortality or only among those with the severe form of HIV infection^25,26^. Our findings differ from cART-era studies because we did not exclude any participants based on severity.

The most plausible mechanism underlying the interaction between age and neurocognitive status in association to mortality may be the age-related exacerbation of co-morbidities such as metabolic disorders and vascular diseases^35–37^. Since the initiation of cART, the prevalence of vascular and metabolic disorders among HIV infected individuals has increased because of improved life expectancy^38^; nearly half of people living with HIV in the United States are aged 50 and older. The synergy between age (and, indirectly, age-related comorbidities) and NCI in HIV probably ensues a vicious cycle. Comorbidities adversely affect the neurocognitive status, which may lead to reduced ability to manage activities of daily life, lower medication adherence, and poorer healthcare utilization, thus further progressing comorbidities^39,40^ Thus age may be acting as a proxy to certain comorbidities.

During domain-specific analyses, we found a stronger negative association between mortality and domain-specific T scores for abstraction and executive functioning, speed of information processing, and motor compared to other domains. The finding is not surprising given the recognition that HIV-related NCI tends to impact frontal-subcortical regions of the brain. To our knowledge, only one other study has conducted an analysis based on subsets of a brief screening instrument that assesses global HIV-related cognitive impairment^27^. Banerjee et al. used the HIV dementia scale (HDS) as a measure of NCI and assessed psychomotor speed, memory, visual-spatial constructional praxis, and executive inhibitory control in relation to mortality. Among the four subset scores, executive inhibitory control (antisaccade subset) was associated with time to death, which is consistent with frontal-subcortical impairment pattern. Lastly, recent studies have found heightened variability within or between neuropsychological test scores to be associated with higher risk of death^41,42^. Such variability may also point to dysfunction related to attention and/or executive functions.

Apart from neurocognitive status, we also found that HIV-infected participants of non-Hispanic ethnicity, with higher viral loads, or lower hemoglobin had a higher hazard of mortality. These findings are adjusted for each other as well as for the neurocognitive status and are consistent with the literature. High plasma viral load and low serum hemoglobin are well-established independent risk factors of mortality in the HIV-infected population. Sempa et al. found a higher hazard of death per unit increase in the log_10_ viral load (HR = 1.63; 95% CI 1.02–2.60)^43^, and a recent study found that viral load was a stronger predictor of mortality compared to CD4 cell count^18^. Lower hemoglobin has also been associated with a higher hazard (HR = 1.32, 95% CI 1.12–1.55) of dying in the HIV-infected population^44^. Multiple studies have found a higher hazard of death among non-Hispanic blacks compared to non-Hispanic whites or overall Hispanics^45,46^. Our findings are somewhat consistent as 72% of NNTC participants were non-Hispanics, and 46% of the non-Hispanics were blacks.

To account for the variability in the course of NCI over time and its reversible nature in relation to mortality, joint modeling was performed. Joint modeling enables the repeated NCI measurements and survival to be modeled while accounting for interrelationships between the two processes^47^. We found that a linear change (increase/decrease) in global T scores is an independent predictor of mortality. To our knowledge, this is the first study to examine repeated measures of NCI in relation to mortality in the HIV-infected population. Our results provide useful predictive information of longitudinal measures of NC status on mortality. The model can be used to predict future survival outcomes as well as future neurocognitive status in terms of T scores when the T scores are known up to the current time (dynamic prediction). Clinicians can use this information for timely detection and appropriate mitigation of NCI based on prediction.

The current study has certain limitations. This is a clinic-based study with a volunteer and predominantly male participation that may limit generalizability. This study, however, is one of its kind, conducted at four centers in the US with information available on a wide range of demographic, behavioral, and clinical and laboratory measures. The study did not exclude anyone based on the presence of other contributing central nervous system (CNS) infections; thus, the NCI may, in part, be imparted by them and not exclusively by HIV infection. However, the prevalence of other CNS infections in the study population was low (9%). An additional limitation was that some variables were incompletely described (e.g., unclear type of hepatitis or cerebrovascular disease), or were based on self-reports (e.g., duration of HIV infection). Among the neurocognitive domains, verbal fluency was a single task domain, which may not be ideal. However, we were limited by the variables available for these secondary analyses, and this is consistent with past work by the NNTC group. Loss to follow up (12%) in the study may have underestimated overall mortality. The final Cox proportional hazards model was not adjusted for additional confounders like depressive symptoms due to missing data. Yet, a sub-analysis of participants with complete data on Beck Depression Inventory-II (BDI-II) scores showed a similar association between NCI and mortality then without BDI-II in the model, although the results cannot be directly compared due to sample size differences. We did not investigate cause-specific mortality, as there was no relevant information available. This may have overestimated mortality in association with NCI. Furthermore, we were not able to assess the potential confounding effects of unmeasured variables, such as comorbidity treatments. Joint modeling was conducted under the assumption of a linear form for the global T score trajectories and censoring being independent of the random effects that may lead to model misspecifications. However, we obtained similar results with splines in the model. The positive average regression coefficient (0.033) for the time variable in the longitudinal sub-model within joint modelling might have been related to practice effects and not to an actual increase in global T scores over the study period. Practice effect is typically stronger between the between 1st and 2nd assessments and less evident in subsequent assessments^48^. Thus, we conducted a sub-analysis excluding the 1st visit. The linear mixed effect model starting at the 2nd visit still generated a positive average regression coefficient (0.022). Lastly, repeated measures were used only for the primary exposure (global T scores), and all other covariates were assessed only at the baseline. Future research may consider including multiple covariates with repeated measures.

## Conclusion

Overall, this study provides evidence that neurocognitive status interacts with age in relation to mortality and thus may have considerable prognostic utility for assessing mortality risk, particularly among older HIV-infected population. The current study identified older HIV-infected population as a group needing special attention for the longevity of life. The finding is substantial given that nearly half of people in the United States living with diagnosed HIV are aged 50 and older and that the neurocognitive status is associated with quality of life. The preliminary findings of the domain-specific analysis may suggest abstraction and executive functioning, speed of information processing, and motor domains to be particularly sensitive in relation to the hazard of mortality. The study further provides evidence that the increase in T scores over time is associated with lower mortality. The joint model provides useful predictive information not only about the hazard of mortality but also future cognitive scores. The study findings may be used to develop a predictive tool and thus may be used for patient-specific timely management strategies and future clinical interventions (lifestyle remedies, optimizing disease and comorbidity management and ARV management). The approach is particularly useful in clinical settings as repeated measures on biomarkers are very commonly generated for monitoring of chronic medical conditions. In conclusion, optimal management (treatment/rehabilitation) strategies, particularly based on the dynamic predictions and targeting not only the neurocognitive status but also age-related co-morbidities, may lead to an improved outcome in the HIV-infected population.

## Supplementary Information

Supplementary Information
