# Supplementary material for: Neurocognitive status and risk of mortality among people living with human immunodeficiency virus: an 18-year retrospective cohort study
Source: Sci Rep. 2021 Feb 12;11:3738. doi: 10.1038/s41598-021-83131-1 (PMC7881128; doi:10.1038/s41598-021-83131-1)
Supplement: Supplementary file 1 — Supplementary Information [file 41598_2021_83131_MOESM1_ESM.pdf]

Neurocognitive Status and Risk of Mortality Among People Living with Human Immunodeficiency Virus: An Eighteen-year Retrospective Cohort Study

Authors and Affiliations

Zaeema Naveed[1], Howard S. Fox[2], Christopher S. Wichman[3], Morshed Alam[3], Pamela May[2], Christine M. Arcari[1], Jane Meza[3], Steven Totusek[2], Lorena Baccaglini[1*]

[1] Department of Epidemiology, University of Nebraska Medical Center, Omaha, Nebraska, USA

[2] Department of Neurological Sciences, University of Nebraska Medical Center, Omaha, Nebraska, USA

[3] Department of Biostatistics, University of Nebraska Medical Center, Omaha, Nebraska, USA

***Corresponding Author**

Lorena Baccaglini, Associate Professor. Department of Epidemiology, College of Public Health, 984355 Medical Center, Omaha, NE 68198-4395

Email: lorena.baccaglini@unmc.edu.

Phone: 402-552-6634.

Supplementary Table S1: Descriptive statistics for battery of individual tests and neurocognitive domains among HIV-infected participants of the National NeuroAIDS Tissue Consortium (NNTC), n=877

Test/Domain	T-score, mean (SD)		
	**Overall**	**Died***	**Survived***
**Global T-score**	41.8 (7.4)	41.6 (7.9)	41.9 (7.2)
**Abstraction/executive functioning**	43.7 (10.4)	43.2 (10.4)	43.9 (10.4)
Trail Making Test, Part B	41.8 (12.4)	41.5 (12.7)	42.0 (12.3)
Wisconsin Card Sorting Test-64, Perseverative Responses	45.9 (12.3)	45.6 (11.6)	46.0 (12.6)
**Speed of information processing**	42.7 (9.8)	42.3 (10.1)	42.9 (9.7)
Wechsler Adult Intelligence Scale-3rd ed. (WAIS-III) Digit Symbol	42.2 (10.4)	41.7 (10.5)	42.4 (10.4)
WAIS-III Symbol Search	43.8 (11.4)	43.5 (11.6)	43.9 (11.3)
Trail Making Test, Part A	42.5 (12.4)	42.1 (12.9)	42.6 (12.1)
**Attention and working memory**	43.2 (8.9)	43.5 (9.5)	43.0 (8.8)
Paced Auditory Serial Addition Task (PASAT)	40.8 (11.4)	41.1 (11.6)	40.8 (11.3)
WAIS-III Letter Number Sequencing	45.7 (9.8)	45.9 (10.1)	45.6 (9.6)
**Learning**	40.3 (8.7)	40.8 (8.9)	40.1 (8.6)
Brief Visuospatial Memory Test-Revised (BVMT-R) Total Recall	41.8 (9.2)	42.7 (9.9)	41.5 (8.8)
Hopkins Verbal Learning Test-Revised (HVLT-R) Total Recall	38.8 (11.4)	38.9 (10.9)	38.8 (11.6)
**Memory**	40.1 (9.7)	40.9 (10.1)	39.7 (9.4)
BVMT-R Delayed Recall	40.7 (11.2)	41.8 (12.2)	41.0 (10.7)
HVLT-R Delayed Recall	39.6 (11.4)	40.1 (11.4)	39.4 (11.4)
**Verbal fluency**	45.7 (11.5)	45.1 (11.9)	45.9 (11.2)
Controlled Oral Word Association Test (COWAT-FAS)	45.7 (11.5)	45.1 (11.9)	45.9 (11.2)
**Motor**	38.9 (11.4)	37.1 (11.4)	39.7 (11.3)
Grooved Pegboard dominant	39.2 (12.5)	36.9 (12.0)	40.3 (12.5)
Grooved Pegboard non-dominant	38.9 (11.5)	37.6 (11.6)	39.6 (11.4)

*The death/survival is based on the study duration i.e., between Jan 2000 – Aug 2018.
